# Supplementary material for: Impact of Surface Trap States on Electron and Energy Transfer in CdSe Quantum Dots Studied by Femtosecond Transient Absorption Spectroscopy
Source: Nanomaterials (Basel). 2023 Dec 22;14(1):34. doi: 10.3390/nano14010034 (PMC10780555; doi:10.3390/nano14010034)
Supplement: Supplementary file 1 [file nanomaterials-14-00034-s001.zip › nanomaterials-2633893-supplementary.pdf]

Supporting information

Impact of Surface Trap States on Electron and Energy Transfer in CdSe Quantum Dots Studied by Femtosecond Transient Absorption Spectroscopy

Hongbin Dou [1,2], Chunze Yuan [1,2,*], Ruixue Zhu [2,*], Lin Li [1,2], Jihao Zhang [1,2] and Tsu-Chien Weng [1,2,*]

[1] School of Physical Science and Technology, ShanghaiTech University, Shanghai 201210, China; douhb@shanghaitech.edu.cn (H.D.); lilin1@shanghaitech.edu.cn (L.L.); zhangjh5@shanghaitech.edu.cn (J.Z.)
[2] Center for Transformative Science, ShanghaiTech University, Shanghai 201210, China
* Correspondence: yuanchz@shanghaitech.edu.cn (C.Y.); zhurx@shanghaitech.edu.cn (R.Z.); wengzq@shanghaitech.edu.cn (T.-C.W.)

Methods:

The fitting and analysis of TAS kinetic lifetime: TAS kinetics at different wavelengths were fitted with multiple exponentials for ODPA-CdSe and OA-CdSe. τ_1 and τ_2 were obtained for ODPA-CdSe. According to time scale, we attributed τ_1 to the time of electrons relaxing to conduction band edge (E_1 state) and τ_2 to the time of electrons back to ground state from E_1 state. Meanwhile, τ_1, τ_2 and τ_3 were obtained for OA-CdSe, which are attributed that τ_1 is the time of electrons relaxing to E_1 state and τ_3 is the time of electrons back to ground state from E_1 state. However, we suggested that τ_2 of OA-CdSe represents the time of electrons transfer from E_1 state to trap state. Thus, the proportion of electrons in different processes is calculated by the amplitude (A_1, A_2 and A_3) from fitting results (Table S4), i.e. the proportion of electrons trapped by trap state is

$$p = \frac{A_2}{A_1+A_2+A_3} \tag{S1}$$

The fitting and analysis of TTET dynamics: To better model the interfacial TTET dynamics, a stretched exponential function was determined as following[32,50] :

$$\Delta A = A_{TTET} \cdot e^{-\left(\frac{t}{\tau_{TTET}}\right)^{\beta}} \tag{S2}$$

For the TTET between QDs and ACA, A_{TTET}, τ_{TTET} and β are obtained from the fitting, representing the amplitude, the stretched lifetime, and the stretching exponent, respectively. Using τ_{TTET} and β, the average weighted lifetime can be calculated by following equation:

$$\langle\tau\rangle = \frac{\tau_{TTET}}{\beta} \cdot \Gamma\left(\frac{1}{\beta}\right) \tag{S3}$$

The corresponding average weighted rate constant <k> can be calculated by:

$$\langle k\rangle = \frac{1}{\langle\tau\rangle} \tag{S4}$$

Furthermore, the rate constant of TTET from QDs-to-ACA can be calculated by the following equation:

$$k_{TTET} = \frac{1}{\langle\tau\rangle} - \frac{1}{\langle\tau_0\rangle} \tag{S5}$$

Where $\langle\tau\rangle$ and $\langle\tau_0\rangle$ represent the average lifetime obtained from stretched exponential fitting of ground state recovery of QDs in the presence and absence of ACA, respectively.

Figures:

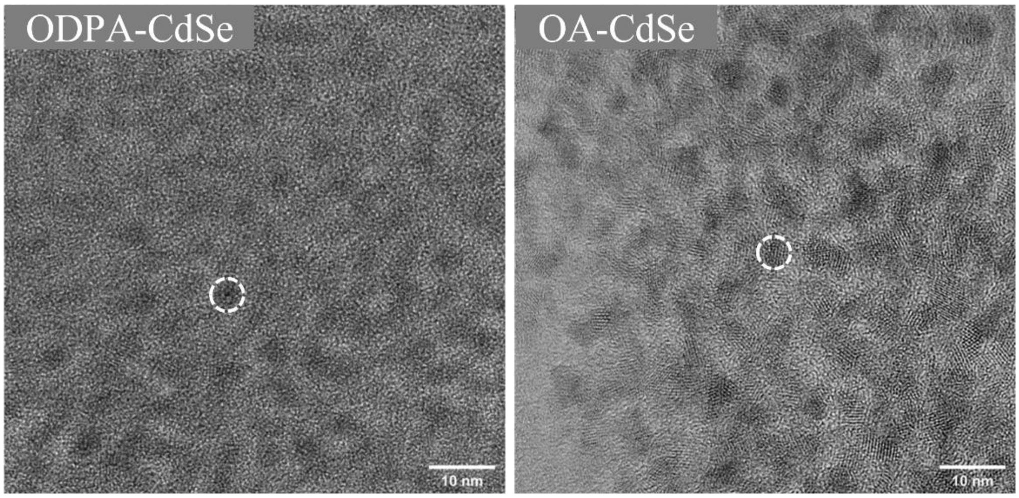

Figure S1. TEM images for ODPA-CdSe and OA-CdSe, both scale bars are 10 nm.

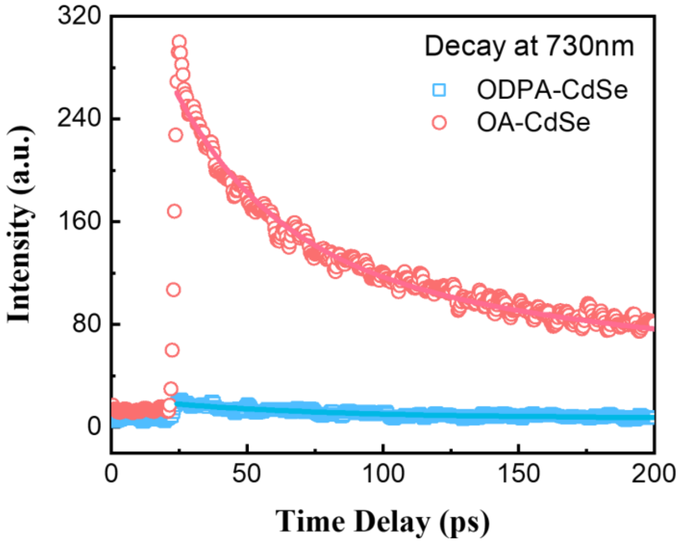

Figure S2. PL kinetics at 730 nm for ODPA-CdSe and OA-CdSe.

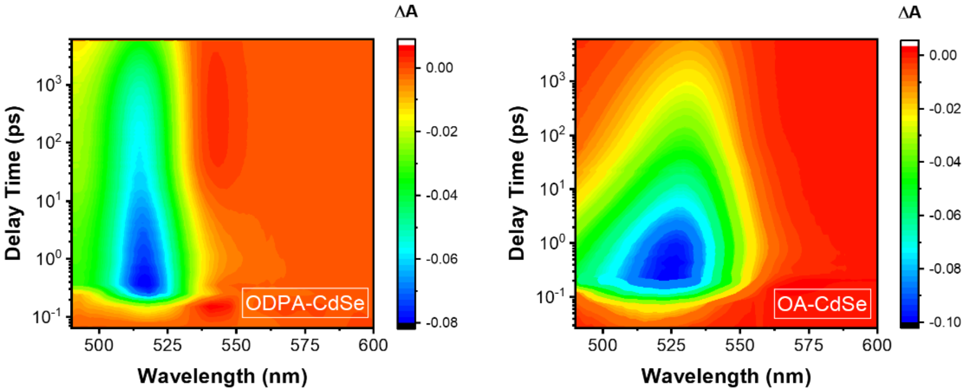

Figure S3. 3D-surface TAS for ODPA-CdSe and OA-CdSe in n-hexane under argon atmosphere, using 480 nm pulsed laser excitation.

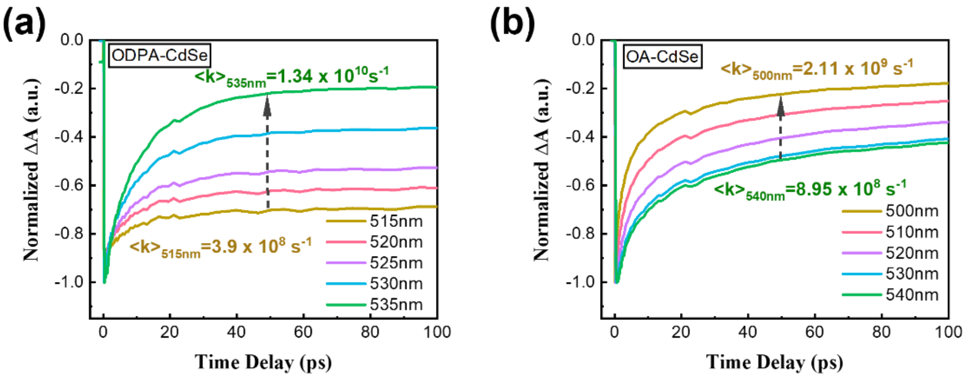

Figure S4. TAS kinetics at different wavelengths for ODPA-CdSe and OA-CdSe in n-hexane under argon atmosphere, using 480 nm pulsed laser excitation.

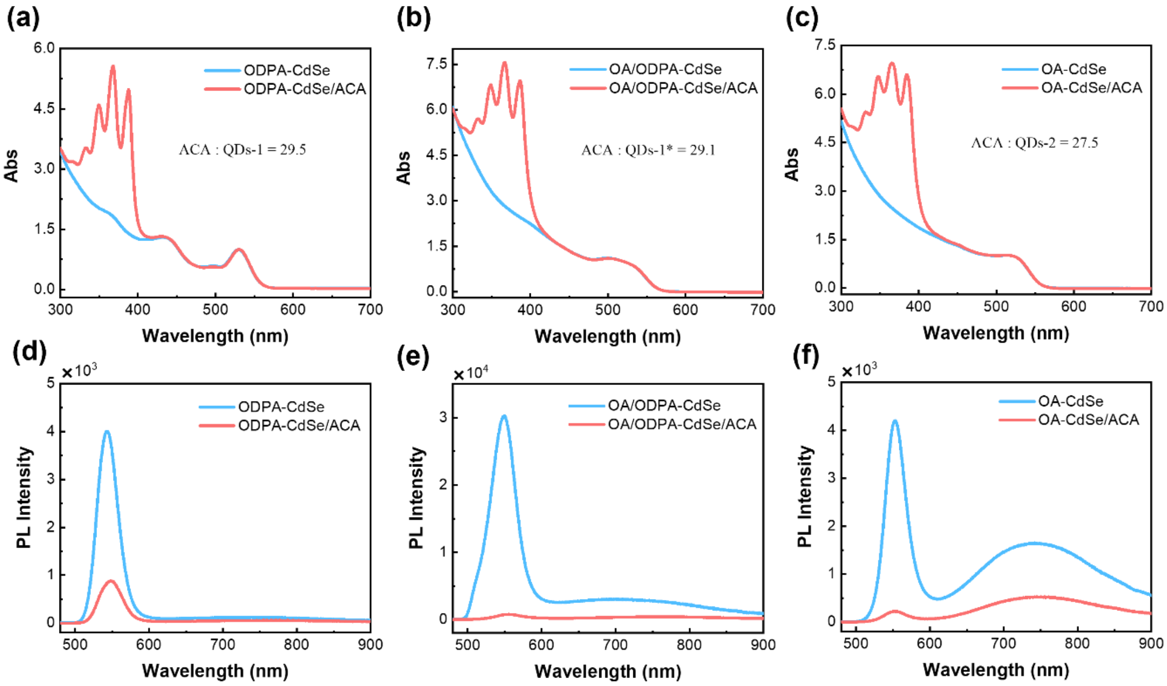

Figure S5. Normalized UV-vis absorption spectra under air atmosphere for (a) ODPA-CdSe, (b) OA/ODPA-CdSe and (c) OA-CdSe with and without ACA, and PL spectra for (d) ODPA-CdSe, (e) OA/ODPA-CdSe and (f) OA-CdSe with and without ACA.

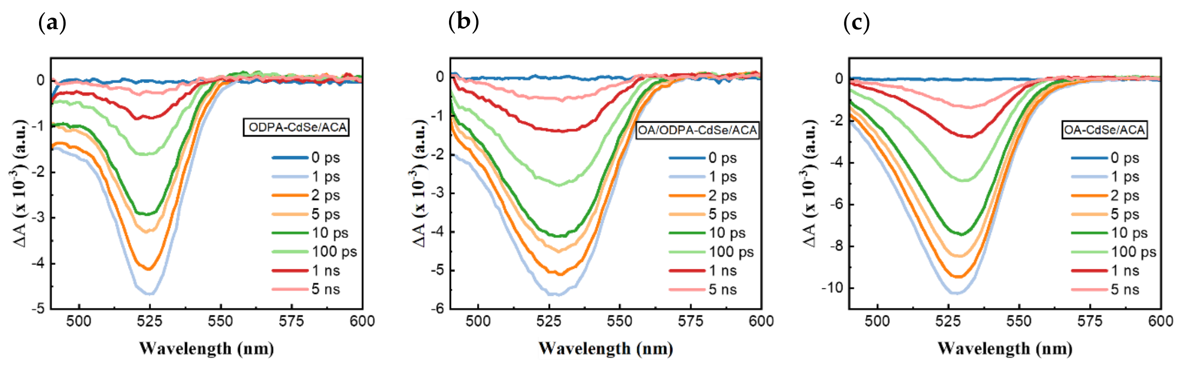

Figure S6. The fs-TA spectra of (a) ODPA-CdSe/ACA, (b) OA/ODPA-CdSe/ACA, and (c) OA-CdSe/ACA in toluene under argon atmosphere. Pump wavelength is 480 nm.

Tables:

Table S1. XPS peak analysis of the Cd 3d regions for ODPA-CdSe and OA-CdSe sample, The peak functions were of Gauss lineshape with 30% Lorentzian character.

Sample	Peak	Binding energy (eV)	FWHM (eV)	Area	% Conc.
ODPA-CdSe	Cd 3d 5/2	404.88	1.01	43189.32	31.28
	Cd 3d 3/2	411.61	1.01	29889.22	28.64
	Cd 3d 5/2 surface	405.38	1.01	28890.12	20.93
	Cd 3d 3/2 surface	412.12	1.01	19991.97	19.16
OA-CdSe	Cd 3d 5/2	405.16	1.01	55146.85	30.89
	Cd 3d 3/2	411.89	1.00	38164.39	28.29
	Cd 3d 5/2 surface	405.66	1.01	38035.17	21.31
	Cd 3d 3/2 surface	412.40	1.00	26322.25	19.51

Table S2. XPS peak analysis of the Se 3d regions for ODPA-CdSe and OA-CdSe sample. The peak functions were of Gauss lineshape with 20% Lorentzian character.

Sample	Peak	Binding energy (eV)	FWHM (eV)	Area	% Conc.
ODPA-CdSe	Se 3d 5/2	53.81	1.48	4674.32	49.65
	Se 3d 3/2	54.41	1.48	3210.16	49.59
	Se 3d 5/2 surface	53.91	1.48	71.35	0.45
	Se 3d 3/2 surface	54.71	1.48	49.01	0.31
OA-CdSe	Se 3d 5/2	53.81	1.15	3272.43	36.14
	Se 3d 3/2	54.61	1.15	2247.39	36.10
	Se 3d 5/2 surface	54.11	1.15	2513.80	16.45
	Se 3d 3/2 surface	54.91	1.15	1726.98	11.30

Table S3. PL kinetic exponential fitting parameters of ODPA-CdSe and OA-CdSe at different wavelengths.

	Wavelength/nm	E_1	τ_1/ns	E_2	τ_2/ns	C
QDs-1	530	22280	17.1	5094	67.9	0
	550	2.219	19.0	0.462	77.6	0
QDs-2	530	3036000	3.8	2609	54.7	0
	550	85.06	4.9	0.2848	65.5	0
	730	261.1	37.9	115.4	308.8	14.85

Table S4. The corresponding exponential fitting parameters of TAS kinetics of ODPA-CdSe and OA-CdSe in n-hexane at different wavelengths.

	Wavelength/nm	A_1	τ_1/ps	A_2	τ_2/ps	A_3	τ_3/ps
ODPA-CdSe	510	-0.0121	7.723	-0.0113	1229	-0.0416	17079
	515	-0.0171	7.525	-0.0119	1171	-0.0465	17079
	520	-0.0214	7.696	-0.00996	1033	-0.0415	17079
	525	-0.0232	8.133	-0.00643	747.6	-0.0278	17079
	530	-0.0211	8.784	-0.00358	379.5	-0.0136	17079
	535	-0.0145	7.711	-0.0045	44.32	-0.0018	17079
OA-CdSe	500	-0.00382	2.757	-0.0213	50.03	-0.00942	4902
	510	-0.0417	3.841	-0.0269	68.69	-0.0158	4902
	520	-0.0418	5.012	-0.0314	84.8	-0.0243	4902
	530	-0.0369	6.104	-0.0309	93.35	-0.03	4902
	540	-0.0259	7.025	-0.022	88.78	-0.0241	4902
	550	-0.0144	7.793	-0.0107	88.32	-0.00702	4902

Table S5. Kinetic traces (Figure 5 (d)(e)(f)) fitting parameter results using Eq. (S2). All data is deduced from TAS kinetics of QDs (ODPA-CdSe, OA/ODPA-CdSe, OA-CdSe) with and without surface anchored ACA in toluene (λ_{ex} = 480 nm).

Wavelength	Samples	τ/ps	β	$\langle\tau\rangle$/ps	$\langle k\rangle/s^{-1}$	$\langle k\rangle_{TTET}/ s^{-1}$
540 nm	ODPA-CdSe	1.107	0.1373	9929	1.0×10^{8}	2.5×10^{9}
	ODPA-CdSe/ACA	0.034	0.1353	377.6	2.6×10^{9}	
	OA/ODPA-CdSe	2127	0.2153	140850	7.1×10^{6}	2.0×10^{8}
	OA/ODPA-	54.78	0.2077	4806	2.1×10^{8}	
	OA-CdSe	17.48	0.1389	132033	7.6×10^{6}	7.5×10^{7}
	OA-CdSe\ACA	44.29	0.1827	12159	8.2×10^{7}	
